# Supplementary material for: Recombinant Subunit Vaccine Candidate against the Bovine Viral Diarrhea Virus
Source: Int J Mol Sci. 2024 Aug 10;25(16):8734. doi: 10.3390/ijms25168734 (PMC11354329; doi:10.3390/ijms25168734)
Supplement: Supplementary file 1 [file ijms-25-08734-s001.zip › ijms-3090317-supplementary.pdf]

Supplementary Materials

Purification of the five E2 proteins.

E2 proteins from subgenotypes BVDV1a, BVDV1b, BVDV1c, BVDV1d, and BVDV1e were purified by IMAC according to section 4.4 of Materials and Methods (Figure S1).

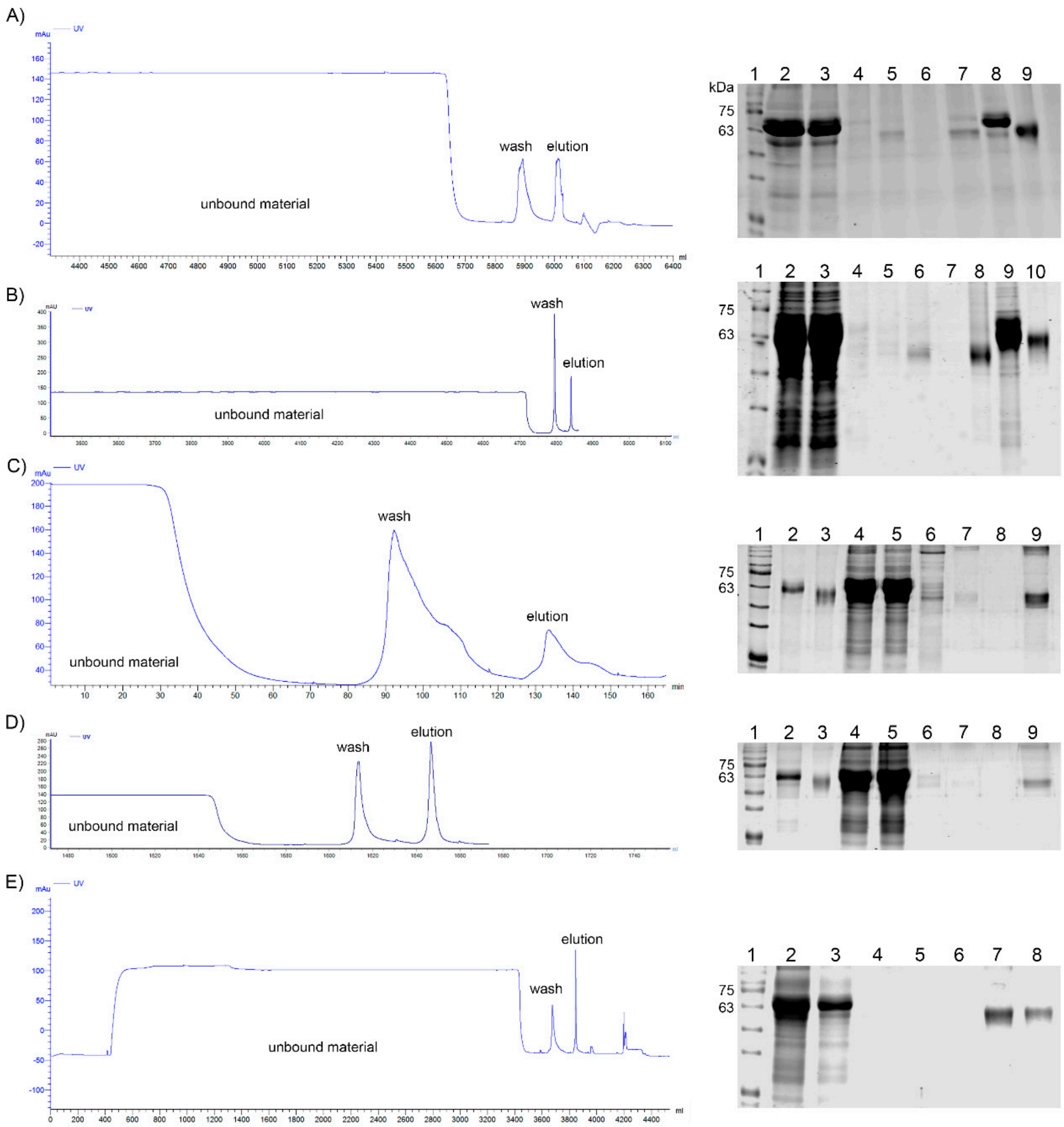

Figure S1. Purification process of E2 proteins. Chromatograms and SDS-PAGE from the purification process (IMAC) of 1aE2 (A), 1bE2 (B), 1cE2 (C), 1dE2 (D), and 1eE2 (E). (A), 1: Molecular Weight Marker AccuRuler RGB Plus prestained protein ladder (MWM) (Maestrogen, Taiwan), 2: initial

sample, 3: unbound material, 4: wash 25 mM imidazole, 5: elution 400 mM imidazole, 6: pass from the concentration step, 7: concentrated protein, 8: negative control, 9: positive control. (B), 1: MWM, 2: initial sample, 3: unbound material, 4: wash, 25 mM imidazole, 5: wash, 40 mM imidazole, 6: elution 400 mM imidazole, 7: pass from the concentration step, 8: concentrated protein, 9: negative control, 10: positive control. (C), 1: MWM, 2: negative control, 3: positive control, 4: initial sample, 5: unbound material, 6: wash 40 mM imidazole, 7: elution 400 mM imidazole, 8: pass from the concentration step, 9: concentrated protein. (D), 1: MWM, 2: negative control, 3: positive control, 4: initial sample, 5: unbound material, 6: wash 40 mM imidazole, 7: elution 400 mM imidazole, 8: pass from the concentration step, 9: concentrated protein. (E), 1: MWM, 2: initial sample, 3: unbound material, 4: wash 40 mM imidazole, 5: elution 400 mM imidazole, 6: pass from the concentration step, 7: concentrated protein, 8: positive control.

Local and rectal temperatures in sheep.

Temperature measurement in sheep from days one to six after the first immunization (Figure S2).

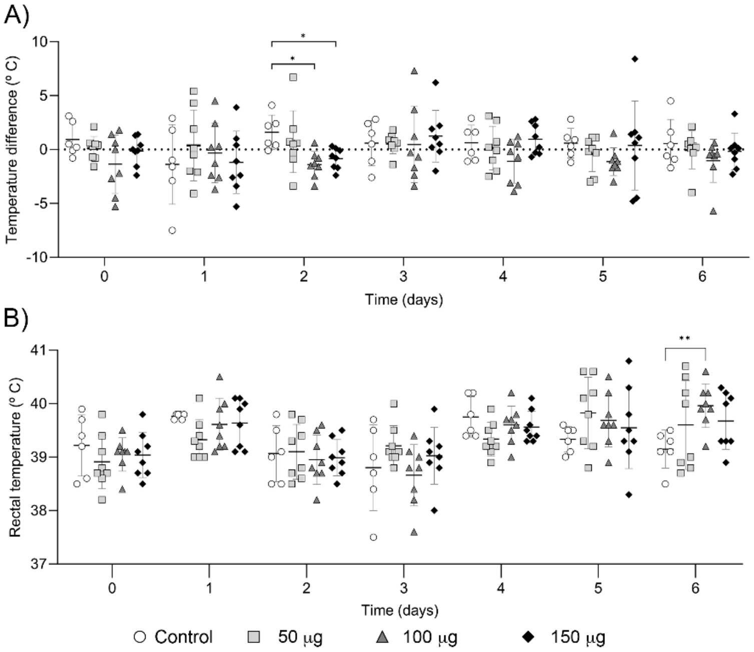

Figure S2. Evaluation of adverse reactions following sheep immunization. (A) Local temperature differences between the right and left buttock six days after the first immunization. (B) Rectal temperature measurements six days after the first immunization. Statistical differences in temperatures among groups at each time were done by the Kruskal-Wallis test, followed by Dunn's multiple comparison test. * $p \leq 0.05$, ** $p \leq 0.01$, *** $p \leq 0.001$, and **** $p \leq 0.0001$.

Rectal temperature in cattle.

Temperature measurement in cattle from days one to three after the first immunization (Figure S3).

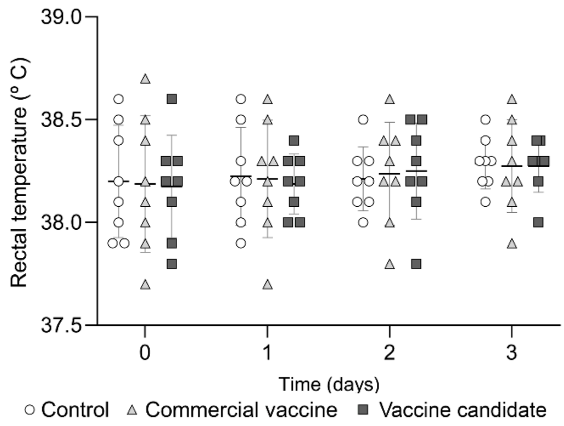

Figure S3. Measurement of rectal temperatures 3 days after the first immunization. Statistics were done each time using the Kruskal-Wallis test, followed by Dunn's multiple comparison test. $p<0.05$. * $p \leq 0.05$, ** $p \leq 0.01$, *** $p \leq 0.001$, and **** $p \leq 0.0001$.
